# NemaFootPrinter: a web based software for the identification of conserved non-coding genome sequence regions between *C. elegans *and *C. briggsae*

**DOI:** 10.1186/1471-2105-6-S4-S22

**Published:** 2005-12-01

**Authors:** Davide Rambaldi, Alessandro Guffanti, Paolo Morandi, Giuseppe Cassata

**Affiliations:** 1 IFOM-FIRC Institute of Molecular Oncology Foundation, Milan, Italy

## Abstract

**Background:**

NemaFootPrinter (Nematode Transcription Factor Scan Through Philogenetic Footprinting) is a web-based software for interactive identification of conserved, non-exonic DNA segments in the genomes of *C. elegans *and *C. briggsae*. It has been implemented according to the following project specifications:

a) Automated identification of orthologous gene pairs.

b) Interactive selection of the boundaries of the genes to be compared.

c) Pairwise sequence comparison with a range of different methods.

d) Identification of putative transcription factor binding sites on conserved, non-exonic DNA segments.

**Results:**

Starting from a *C. elegans *or *C. briggsae *gene name or identifier, the software identifies the putative ortholog (if any), based on information derived from public nematode genome annotation databases. The investigator can then retrieve the genome DNA sequences of the two orthologous genes; visualize graphically the genes' intron/exon structure and the surrounding DNA regions; select, through an interactive graphical user interface, subsequences of the two gene regions. Using a bioinformatics toolbox (Blast2seq, Dotmatcher, Ssearch and connection to the rVista database) the investigator is able at the end of the procedure to identify and analyze significant sequences similarities, detecting the presence of transcription factor binding sites corresponding to the conserved segments. The software automatically masks exons.

**Discussion:**

This software is intended as a practical and intuitive tool for the researchers interested in the identification of non-exonic conserved sequence segments between *C. elegans *and *C. briggsae*. These sequences may contain regulatory transcriptional elements since they are conserved between two related, but rapidly evolving genomes. This software also highlights the power of genome annotation databases when they are conceived as an open resource and the possibilities offered by seamless integration of different web services via the http protocol.

**Availability**: the program is freely available at

## Background

Comparative genomics is a powerful bioinformatics methodology for the identification of conserved genomic DNA segments between two related organisms [1]. Alignment of DNA sequences from different species provides an effective tool to decode genomic information, based on the assumption that functional sequences tend to diverge at a slower rate than non-functional sequences. By comparing the genomic sequences of species at different evolutionary distances, it is possible, besides identifying coding sequences, to recognize conserved non-coding sequences with a potential regulatory function, and determine which sequences are unique for a given species. This procedure is called Phylogenetic Footprinting [2,3]. Alignment algorithms optimize these comparisons so that the regions, that diverge slowly, can be anchored together and highlighted against a background of more rapidly evolving DNA, that is devoid of any functional constraints [4]. On a broader view, the identification of non-exonic Conserved Sequence Elements tags associated with human disease-related genes may open new venues for the interpretation of experimental data [5].

Although *C. elegans *and *C. briggsae *are almost identical in morphology and development [6], their genomes have diverged. Several estimates suggest that separation of the two species occurred 23–40 million years ago [7]. Conservation of DNA sequences is confined largely to protein-coding regions and short flanking sequences; functional conservation between these two species has also been demonstrated by rescue experiments of mutant phenotypes via DNA-mediated transformation [8].

We developed an interactive web-based and user friendly software to help the researchers in the identification of conserved non-coding sequence regions between the genomes of the two nematodes *C. elegans *and *C. briggsae*, starting from a bio-computational project focused on identification of conserved segments in a single pair of orthologous genes.

## Implementation

The program developed here is a research tool; hence the design has been sometimes bound to the functionality, in order to optimize the speed and easiness of interaction between all the components. A careful planning of all the required modules has been achieved, however we have used system analysis and design techniques for the most complex parts of this development project.

Documentation has been targeted both at the user with a web page , and to the programmer/maintainer of the software, with internal documentation on the scripts. We always relied on feedback from the interested scientist in designing the user interface and ameliorating the software functionality. From the programming language point of view, we adopted standard solutions that were fit to the problem (bioinformatics development).

The following components have been used to develop the web application:

1) A local mirror of Wormbase [9] database under mySQL. The genome annotation information in Wormbase is maintained in General Feature Format (GFF). GFF is a text-based format for the transfer of genome information, allowing genome researchers to develop tools and have them tested without having to maintain a complete feature-finding system. Documentation on this format is available at 

2) An EnsMart table to search orthologs. EnsMart [10] is a data retrieval tool that generates lists of biological objects (e.g. genes, SNPs) from data held in the Ensembl database. EnsMart uses the BioMart system .

3) A Perl web interface used to retrieve gene and sequences, mask exons, launch analysis tools, and generate gene images (GD libraries). Other web based technologies are implemented inside the Perl code: cursor coordinates capture and client-side image maps are implemented in html and JavaScript.

4) A graphical user interface implemented in Java (Swing Applet) for interactive selection of sub-sequences. Using HTTP POST and GET method, the Applet is able to communicate with other elements of the system.

5) A collection of locally compiled C++ software: **dotmatcher **and **extractseq **(EMBOSS package), **blast two sequences **(NCBI), **ssearch **(part of the FASTA program suite written by William Pearson), **blastz**.

6) An User Agent LWP connection (Perl library) to send subsequences and blastz alignment to a transcription factor database web server (**rVista**)

## Results and Discussion

**Figure 1:**
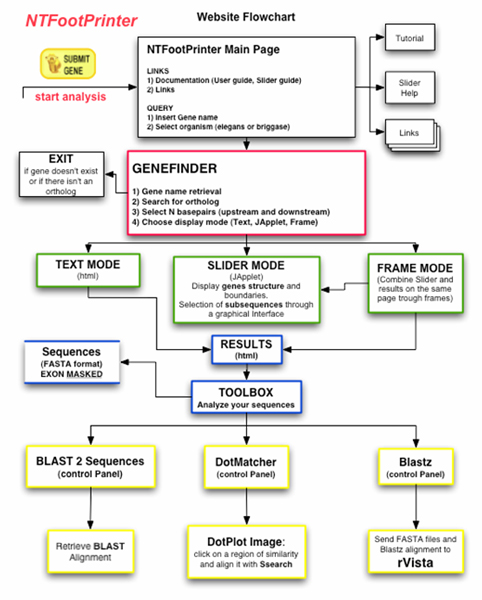
**Web Interface Scheme. NemaFootPrinter scheme: **starting from gene name submission ('Start analysis' on the top-left of the scheme), gene name and organism are submitted to the GENEFINDER script (red-border box) that verifies if the given gene has an ortholog and displays the clone name. GENEFINDER also allows not-interactive selection of *n *base pairs upstream and downstream of the given genes. After gene-name retrieval the user can choose a display mode (green-border boxes): **TEXT-MODE **allows non-interactive selection of subsequences; the **SLIDER-MODE **uses an Applet Java to select interactively sub-sequences; the **FRAME-MODE **combines the slider and the result page on two horizontal frames. On the **RESULTS **page (blue-border boxes) users can find images of genes structure and boundaries generated on the fly, links to the sequences (FASTA format) and links to a series of bioinformatics tools (yellow-border boxes): **BLAST 2 sequences **is used for a first screening of the two sequences for similarities; **Dotmatcher **generates a dot plot and associate an image for direct graphical visualization of regions of similarity. By clicking on a point into the Dotmatcher image and extending the selection for *n *base pairs, it is possible to align small regions with the *Smith and Waterman *algorithm. One can then send Fasta files and Blastz alignments to the **rVista **server (the two subsequences and the relative **Blastz **alignment generated on the local server). Through this public database it is possible to identify the transcriptional regulatory elements, if any, associated with conserved subsequences.

The main functional flow of the software can be summarized as follows (Figure 1): Starting from a *C. elegans *or *C. briggsae *gene name or identifier, the software identifies the putative ortholog (if any), based on information derived from one of the EnsMart datamart tables [10]. In this first step, the user has the opportunity to start from either an identifier ('gene model') or from a 'common gene name', following the *CGC *(*Caenorhabditis Genetics Center*) genetic nomenclature [11].

**Figure 2:**
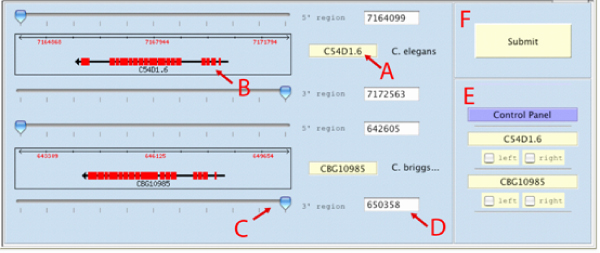
**Select subsequences. **CBrothersSlider.java is a small application written in Java to interactively display gene structures with intron/exon structure and to select subsequences. The interface display clone identifiers (A) and gene images generated "On-the fly" (B). Shifting the sliders (C) or submitting directly chromosome coordinates (D), the user is able to select a subsequence. A small control panel (E) can be used to select only the region on the left of the gene (*left *checkbox) or on the right of the gene (*right *checkbox); if both the checkboxes are selected, the application selects only the gene sequence. After sequence manipulation, using the Submit button (F), user can post the selected subsequence coordinates to the main script that generates new FASTA files and display the Results page.

The user can select a display mode:

1. **TEXTMODE**: html output only

2. **SLIDER MODE**: graphical visualization with a Java Applet (Figure 2)

3. **FRAME MODE**: both results and slider on the same web page using frames

The software retrieves the sequences of the two orthologous genes from the local database. At this step, exons of the two orthologous genes are masked, since similarities between conserved coding regions are not interesting for our purpose. After sequence retrieval, the software generates *'On-the fly' *an image of the gene structures with associated intron / exon structures.

A Java applet (Figure 2) displays gene structures and neighbourhoods, the user can select subsequences. The same operation can be performed in text mode. After this step, the user can start sequence analysis from the results page.

On the results page the investigator can identify and analyze sequence similarities with a number of tools:

•** Pairwise Blast**[**12**]**: **While the standard BLAST program is widely used to search for homologous sequences in nucleotide and protein databases, it is necessary to compare only two sequences to ascertain their similarity or common features. In such cases, searching the entire database would be unnecessarily time-consuming. 'BLAST 2 Sequences' utilizes the BLAST algorithm for pairwise DNA-DNA or protein-protein sequence comparison.

**Figure 3:**
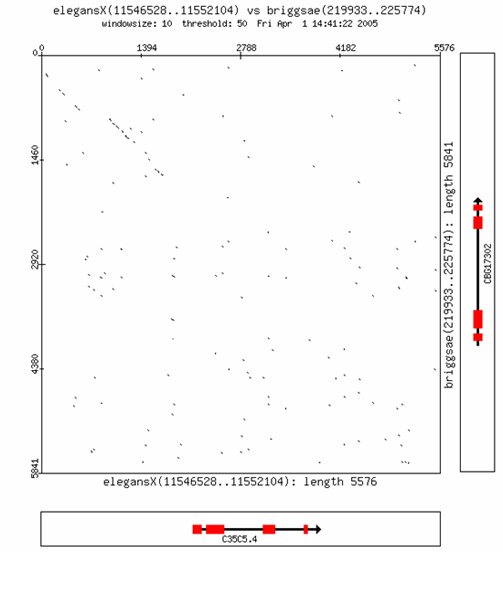
**dotmatcher. **A Dotplot image: *C. elegans *and *C. briggsae *sequences are placed on the axes. The gene structure generated on the fly help the Investigator to orient itself in the rectangular image. The top of the image shows parameters given by the investigator (windowsize and threshold). This web images are clickable by the user, the single click is extended in both directions for *n *base pairs. Both segments (one for *C. elegans *and one for *C. briggsae*) are sent to the Ssearch control page, in order to align segments with the Smith and Waterman algorithm.

•** Dotmatcher**[**13**]**:** Dotplot is a graphical representation of the regions of similarity between two sequences. The two sequences are placed along the axes of a rectangular matrix and (subject to threshold conditions) wherever there is equality between the sequences a dot is placed on the image. Where the two sequences have substantial regions of similarity, many dots align to form diagonal lines. It is therefore possible to glance at local regions of similarity, as these will show diagonal lines (Figure 3). In this version of Dotplot the user can control **window size**, **threshold **and **which strand to align**: a small subroutine in the web page named '*strand-helper*' helps in choosing the best configuration (align plus strand against plus, minus strand against minus, plus against minus, etc.). Even if the software selects a default strand configuration, the user can manually choose another strand mode. On the web page, text boxes give the exact position (relative to the effective sequence length) of the cursor on the X and Y-axis; this helps the user in choosing the sequence stretch of interest.

• **Ssearch **[**14**]**:** Ssearch uses Pearson's implementation of the method of Smith and Waterman [15] to search for similarities between one sequence (the query) and any group of sequences of the same type (nucleic acid or protein). After the Smith-Waterman score for a pairwise alignment is determined, Ssearch uses a simple linear regression against the natural log of the search set sequence length to calculate a normalized z-score for the sequence pair [16]. The distribution of the z-scores tends to closely approximate an extreme-value distribution; using this distribution, the program can estimate the number of sequences that would be expected to produce a z-score greater than or equal to the z-score obtained in the search. This is reported as the E() score. When all of the search set sequences have been compared to the query, the list of best scores is printed. In our implementation Ssearch is used for aligning two subsections isolated from the Dotplot output.

• **BlastZ**[**17**]**and rVista **[**18**]**: BlastZ **computes local alignments for sequences of any length based on the assumption that the input sequences are related and share blocks of high conservation that are separated by regions that lack similarity and vary in length. Regions of homology are displayed collinear only to the reference sequence, while the order and orientation of the conserved elements is not necessarily the same in the second sequence.

Identifying transcriptional regulatory elements represents a significant challenge in annotating genomes. Our bioinformatics procedure has been transparently connected trough the http protocol to a computational tool, **rVista**. rVista is aimed at high-throughput discovery of *cis*-regulatory elements, combining clustering of predicted transcription factor binding sites (TFBSs) and maximizing the identification of functional sites.

A continuous exchange of ideas and information about this software and its interface occurred between the software developers and the nematode researchers. This user feedback has hence been fundamental in tailoring the graphical interface in functions of his needs.

A PAIRWISE alignment performed with **blast2seq **(blast two sequences), a heuristic algorithm (BLAST), is used for fast comparison of two sequences.

**Dotmatcher **uses a simple but slow algorithm; for this reason we have chosen this as a tool to verify the alignments identified in the first step. When two regions of similarity have been found, this similarity can be quantified with the **Smith and Waterman algorithm **(best local alignment). This is the most sensitive method available for pairwise sequence comparison, but it works slowly, therefore it is more appropriate for in depth analyses than primary searches.

The last component of the toolbox is a transparent connection through the http layer to a database search tool (**rVista**) focused on the identification of transcription factor binding sites related to conserved sequence elements. Continuous update of the transcription factors for vertebrates and, in particular, for nematodes is thus guaranteed. Local execution of **blastz **algorithm permits a fast genome sequence alignment, while the remote server (**rVista**) performs transcription factor binding site analysis.

Finally, in order to test the quality of the software, we performed the following experiment: Natarajan and colleagues [19] have recently determined enhancer elements in the *C. elegans *gene encoding for a beta catenin homologue: *bar-1*. By classical promoter analysis (creating transgenic lines bearing deletion constructs of the promoter region) they were able to identify two different Cis acting elements [19]. In a second step, by alignment, they show that these enhancers/elements are conserved in *C. briggsae*. By simply using our software we were able to confirm all the elements described in this work without any molecular biology experimentation needed (data not shown). With the help of NemaFootPrinter, from now on, researchers will "first" use our software and "then" test the results by creating just a few transgenic strains in order to just "test" the results and not to "identify" the regions by tedious *in vivo *experiments.

### Other tools for performing comparative genomics

CisHorto, another tool for transcription-factor identification [20], uses Position Weight Matrices and a user-provided ungapped multiple alignment to predict new transcription factors binding sites. Our software adopts a simpler strategy for the identification of putative transcription factor sites, based on sequence similarity and exon masking to highlight similarities between non-coding regions.

CSTminer [21] is an user-friendly tool for generic identification of coding and noncoding conserved sequence tags through cross-species genome comparison, that uses an original algorithm to identify statistically significant conserved blocks and assess their coding or noncoding nature through the measure of a "coding potential score". We focused our development specifically on nematode genetics, leaving to the final user a high degree of interactivity and exploration for the identification of conserved sequence regions.

## Conclusion

Genome annotation databases such as Wormbase [22] or EnsEMBL [23] have a fundamental role in modern biological research and they also offer a platform to bioinformatics development. We have presented a simple web-based software aimed to identify conserved functional segments outside exons (putative new gene expression control elements) through comparative genomics between the nematodes *C. elegans *and *C. briggsae *[24]. With this project we have highlighted that a specific bioinformatics project can be realized by a sound integration of genome database mirrors, local development and transparent integration with remote resources [25]. Where speed and robustness were needed, we relied on local mirroring of databases and development of software modules, but when other resources already solved the task we integrated seamlessly calls to remote services through the http protocol. As a result, a new resource, aimed at solving a specific biological problem is now freely available at . We have also demonstrated that usability and functionality in bioinformatics development can be achieved only through a strong and continued feedback from the scientist/user.

## Availability and requirements

**Project name: ***NemaFootPrinter*

**Project home page: **

**server side**: UNIX type platforms

**client side**: Any operating system

**Programming language: **SQL, Perl, Java

Other requirements:

The web-based application was tested and is compatible with the more common Internet browser. For the Slider Applet the user must have a Java Virtual Machine installed and configured on the client. User without Java can use the TEXT MODE to analyze genes. Even the older *text*-*only *browsers like 'lynx' are compatibles with the software (obviously text-only browser must use the TEXT MODE display). For more compatibility information check the help page: 

## Authors' contributions

All authors contributed to the development of NemaFootPrinter.

DR wrote most parts of the NemaFootPrinter core, the CGI scripts, the Java applet and the database handlers. AG was responsible of the main bioinformatics programming strategy.

PM was respnsible of biological applied aspect.

GC made the scientific supervision and interface design. All authors drafted the manuscript and approved the final version.
